# Retroviral prototype foamy virus intasome binding to a nucleosome target does not determine integration efficiency

**DOI:** 10.1016/j.jbc.2021.100550

**Published:** 2021-03-18

**Authors:** Randi M. Kotlar, Nathan D. Jones, Gayan Senavirathne, Anne M. Gardner, Ryan K. Messer, Yow Yong Tan, Anthony J. Rabe, Richard Fishel, Kristine E. Yoder

**Affiliations:** 1 Cancer Biology and Genetics, The Ohio State University College of Medicine, Columbus, Ohio, USA; 2 The Ohio State University Comprehensive Cancer Center, Columbus, Ohio, USA

**Keywords:** nucleosome, retrovirus, integration, protein–protein interaction, enzyme catalysis, prototype foamy virus, bp, base pair, CCD, catalytic core domain, CIC, conserved intasome core, CTD, carboxyl terminal domain, ΔCTD, deletion of the CTD, FL, full length, FRET, Förster resonance energy transfer, IN, integrase, NED, amino terminal extension domain, NPS, nucleosome positioning sequence, NTD, amino terminal domain, ΔNTD, deletion of the NED and NTD, PAGE, polyacrylamide gel electrophoresis, PFV, prototype foamy virus, TL, nucleosomes treated with trypsin to remove the histone tails, vDNA, viral DNA

## Abstract

Retroviral integrases must navigate host DNA packaged as chromatin during integration of the viral genome. Prototype foamy virus (PFV) integrase (IN) forms a tetramer bound to two viral DNA (vDNA) ends in a complex termed an intasome. PFV IN consists of four domains: the amino terminal extension domain (NED), amino terminal domain (NTD), catalytic core domain (CCD), and carboxyl terminal domain (CTD). The domains of the two inner IN protomers have been visualized, as well as the CCDs of the two outer IN protomers. However, the roles of the amino and carboxyl terminal domains of the PFV intasome outer subunits during integration to a nucleosome target substrate are not clear. We used the well-characterized 601 nucleosome to assay integration activity as well as intasome binding. PFV intasome integration to 601 nucleosomes occurs in clusters at four independent sites. We find that the outer protomer NED and NTD domains have no significant effects on integration efficiency, site selection, or binding. The CTDs of the outer PFV intasome subunits dramatically affect nucleosome binding but have little effect on total integration efficiency. The outer PFV IN CTDs did significantly alter the integration efficiency at one site. Histone tails also significantly affect intasome binding, but have little impact on PFV integration efficiency or site selection. These results indicate that binding to nucleosomes does not correlate with integration efficiency and suggests most intasome-binding events are unproductive.

Following entry to a cell, the retroviral enzyme reverse transcriptase copies the viral genomic RNA to a linear double-stranded cDNA (1). Then retroviral integrase (IN) covalently joins the cDNA 3′ ends to host DNA. The integration reaction is faithfully recapitulated *in vitro* by complexes assembled from recombinant IN and oligomers mimicking the viral DNA ends (vDNA), termed intasomes (2). The IN of prototype foamy virus (PFV) has four domains: an amino terminal extension domain (NED), an amino terminal domain (NTD), a catalytic core domain (CCD), and a carboxyl terminal domain (CTD) (3). Structural studies of PFV intasomes revealed a tetramer of IN arranged with two catalytically active inner protomers and two structurally important outer protomers (2, 4, 5). All IN domains of the PFV intasome inner protomers have been visualized, but only the CCDs of the outer protomers have been resolved. In addition to the IN tetramer observed for PFV, retroviral intasomes may also be an octamer of IN, in the cases of mouse mammary tumor virus and Rous sarcoma virus, or a hexadecamer of IN, as seen with Maedi visna virus (6, 7, 8). Central to the higher order multimers is a conserved intasome core (CIC) structurally similar to the PFV IN tetramer (9). The CIC suggests that the PFV intasome may serve as a model for intasomes of other retroviruses.

The natural target for retroviral integration is eukaryotic chromatin, which consists of DNA packaged into arrays of nucleosomes. A nucleosome is ∼147 bp of DNA wrapped around an octamer of histone proteins H2A, H2B, H3, and H4. Nucleosome DNA and histones display symmetry around a central dyad (10). Mononucleosomes may be assembled from recombinant histones and a nucleosome positioning sequence (NPS) DNA (11). Natural NPS DNAs display rotational and/or translational shifting on the histone octamer particularly under conditions of physiological ionic strength (12). A synthetic NPS termed 601 was selected *in vitro* to maintain stable positioning over a wide range of ionic strengths, even below physiological conditions, yet has been shown to retain physiologically relevant nucleosome dynamics *in vivo* (13, 14). The NPS DNA sequence is numbered –73 to +73 with the 0 base pair (bp) at the dyad and the distal ends termed entry–exit regions (Fig. 1*A*). 601 NPS DNA binds tightly to a histone octamer at the dyad while the entry–exit regions may transiently unwrap (15, 16).

**Figure 1:**
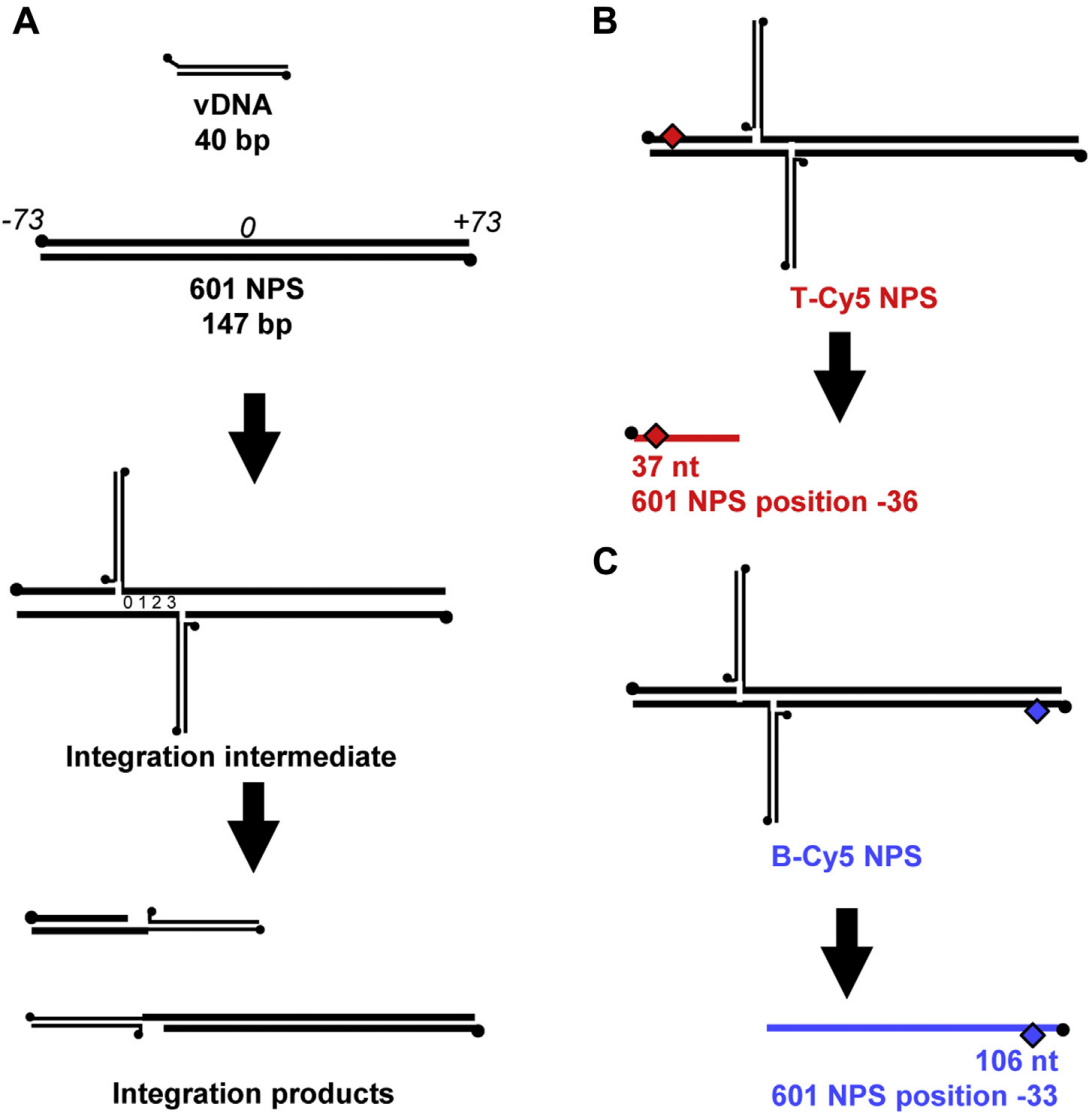
**PFV integration into a linear DNA target.***A*, illustration of PFV concerted integration to 601 NPS DNA, not drawn to scale. PFV 40 bp vDNA (*thin lines*) in the context of an intasome is added to 601 NPS DNA (*thick lines*) wrapped around a histone octamer. The 601 NPS is 147 bp numbered –73 to +73 with 0 at the central dyad. Integration of the vDNA to the NPS yields an integration intermediate. The two PFV strand transfer events are separated by 4 bp of target DNA, indicated by numbers 0–3. For a single concerted integration, two integration products are formed. When analyzed by native gel electrophoresis, the products appear as two bands. Each product is one vDNA, a fraction of the NPS DNA, and a four base gap. *Black circles* indicate 5′ ends. *B* and *C*, the NPS DNA may be fluorescently labeled (*red* or *blue diamond*) on either the top (T-Cy5 NPS) or bottom (B-Cy5 NPS) strand. The integration strand transfers introduce nicks in the target DNA. Each nick is immediately adjacent to a point of joining. Denaturing gel electrophoresis isolates the NPS DNA fragment that is not joined to the vDNA. As an example, concerted integration could yield a 37 nt fragment and a 106 nt fragment (37 nt + 106 nt + 4 nt space between strand transfers = 147 nt, the length of 601). Based on the 601 numbering, the vDNA is joined to T-Cy5 NPS DNA at –36 and B-Cy5 NPS -33. Integration position 0 is at 601 NPS position –36.

For decades retroviral integration has been known to favor bent DNA, particularly in the context of chromatin (17, 18, 19). One possible model for intasome interaction with nucleosomes was based on DNA-binding protein searches of chromatin (20). Some transcription factors or DNA damage sensors slide on transiently unwrapped entry–exit regions of nucleosomes to search for a binding site (15, 16, 21, 22, 23). PFV intasomes are also capable of performing one-dimensional diffusion on DNA and search 1.6 kb while in continuous contact with naked linear DNA (24). Conceivably, PFV intasomes could slide on transiently unwrapped NPS DNA to an integration site by a mechanism similar to other DNA-binding proteins. However, nucleosomes with engineered histone posttranslational modifications (PTMs) that induce NPS unwrapping had no effect on integration efficiency (20). This data suggests that intasomes do not integrate to nucleosome-bound DNA by sliding on exposed entry–exit regions. A different model is derived from an elegant structure of the PFV intasome bound to a nucleosome revealing the CTD of an inner IN protomer bound to an H2A tail (5). Other retroviruses, such as human immunodeficiency virus (HIV-1) or murine leukemia virus (MLV), use host proteins to tether their integration complexes to nucleosomes by binding IN and histone PTMs (25, 26, 27, 28). PFV intasomes appear able to bind directly to nucleosomes to complete integration into wrapped DNA.

Structural studies indicated a strategy to position PFV IN protomers at either the inner or outer intasome subunits by compensatory point mutations (5). PFV IN(K120E) and PFV IN(D273K) direct IN to the catalytically active inner or inactive outer positions, respectively. Deletion of the NED, NTD, or CTD domains may be engineered at the outer protomers by the IN(D273K) mutation (5). The IN(D273K) truncation mutants can be assembled with full length (FL) IN(K120E) and vDNA to generate enzymatically active PFV intasomes (5, 29). Biochemical assays revealed that the outer protomer NED, NTD, and CTD domains are not required for catalysis (29, 30). Indeed, deletion of the outer protomer CTDs led to increased stability of the PFV intasomes without affecting binding to a supercoiled plasmid target DNA (29). Deletion of the amino terminal domains of the outer protomers showed minimal effects on intasome stability. Whether the outer protomer NED, NTD, and CTD domains play any meaningful roles in PFV integration to chromatin is largely unknown.

The effects of outer protomer truncation mutations during integration to nucleosomes have not been explored at ionic strength conditions in the physiological range. Here we assembled fluorescently labeled human mononucleosomes as well as PFV intasomes with FL IN, deletion of the IN amino terminal NED and NTD domains (ΔNTD), or deletion of the IN CTD (ΔCTD) at the outer protomers. Integration efficiency was quantified and integration sites were mapped to the 601 nucleosome structure (PDB: 3LZ0, (31)). PFV intasomes integrate to 601 nucleosomes in four clusters at exposed helices. Deletion of the outer PFV IN domains had minor effects on integration efficiency or site selection. These effects were mostly seen at a single integration cluster and only observed in the presence of 100–150 mM NaCl. To analyze binding of the truncation mutants to nucleosomes, PFV intasome vDNA was biotinylated and examined by streptavidin affinity precipitation. PFV IN(ΔCTD) intasomes displayed significantly less binding to nucleosomes. Nucleosomes were treated with trypsin to evaluate the roles of histone tails on integration efficiency and binding at physiological ionic strength conditions. While removal of histone tails showed significant effects on intasome binding, there were no significant differences in overall integration efficiency. The minor differences of integration efficiencies did not correlate with significant differences in binding, suggesting that PFV intasome binding to a target is not rate determining for catalysis.

## Results

### Kinetics of PFV intasome integration into nucleosomes

Purified PFV intasomes are a tetramer of IN and two vDNAs (2). These complexes readily perform integration to target DNA *in vitro* (3). Concerted integration is the covalent joining of each vDNA to target DNA in a reaction termed strand transfer. The two points of joining are separated by 4 bp of target DNA numbered 0–3 (Fig. 1*A*). Concerted integration to a linear target DNA generates two fragments, each consisting of one vDNA and a fraction of the target DNA. A four-nucleotide single-strand DNA gap is at the junction of vDNA and target DNA.

Recombinant mononucleosomes include 147 bp of linear NPS DNA wrapped around a histone octamer. The 601 NPS DNA is numbered –73 to +73 with the central base pair numbered 0 (Fig. 1*A*). Integration products from nucleosome substrates are two linear DNA fragments of vDNA ligated to target DNA. Fluorophore labeling of either the top or bottom strand of the 601 NPS allows identification of the points of joining on each strand (Fig. 1, *B* and *C*). Alternatively, intasomes may join only one vDNA to the target generating a Y structure DNA product. These half-site integration products are not likely to be physiologically relevant but may appear during integration *in vitro*.

**Figure 2:**
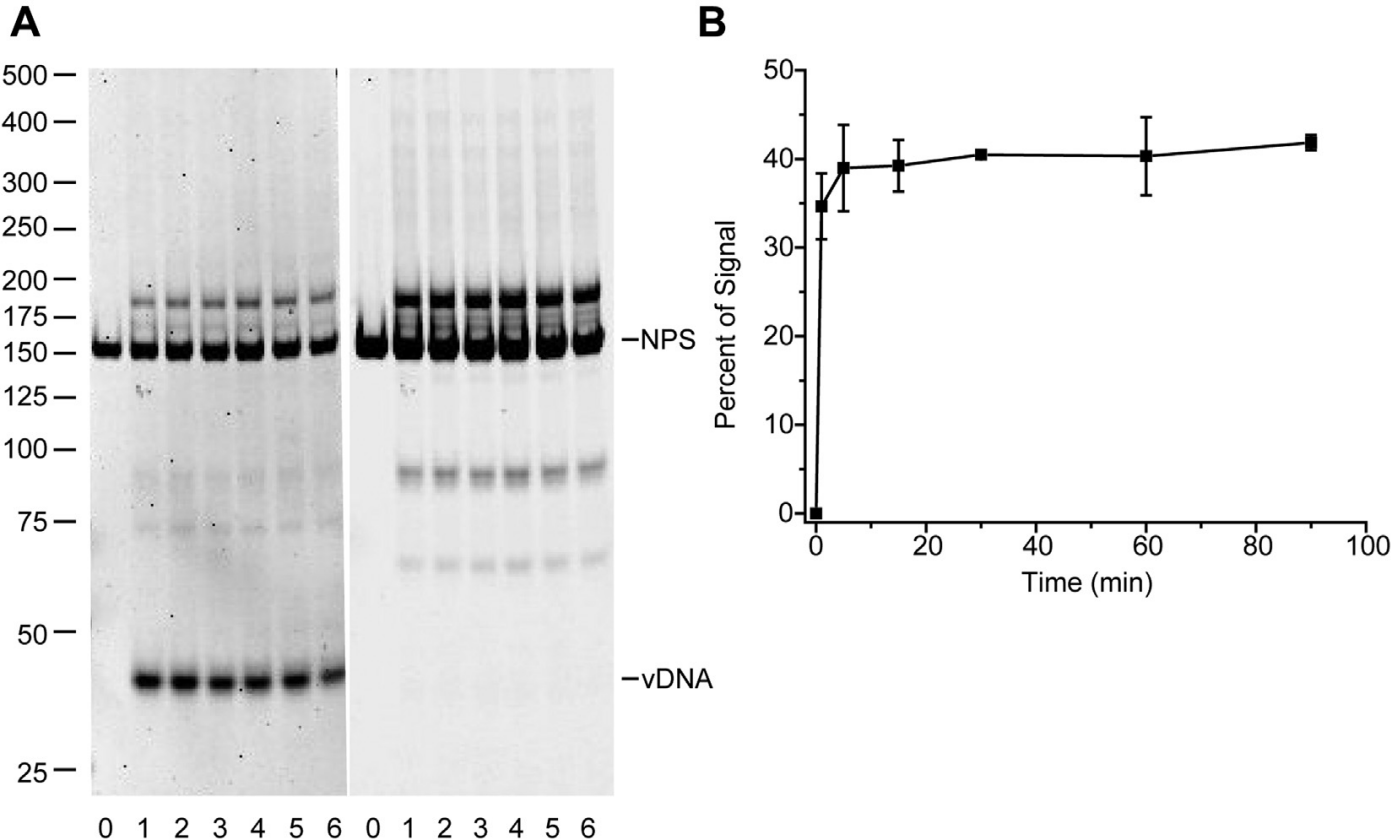
**Time course of PFV integration to a nucleosome target**. *A*, PFV intasomes with a 40 bp vDNA (vDNA) were incubated with 147 bp Cy5 labeled 601 nucleosomes (NPS). Reaction products were separated by native PAGE stained with ethidium bromide. *Left*, ethidium bromide image. *Right*, Cy5 image. Lane 0 is 601 nucleosome substrate only. Lanes 1–6 are 1, 5, 15, 30, 60, and 90 min incubation times. DNA ladder sizes are on the left in bp. *B*, quantitation of integration over time with Cy5 labeled nucleosomes. The total Cy5 fluorescence in each lane was measured. The percentage of signal is the fluorescent signal excluding the substrate band. Error bars indicate the standard deviation between three independent experiments with at least two PFV intasome and nucleosome preparations.

Integration assays with PFV intasomes have been performed at 37 °C for 30–60 min (2, 30). Our previous kinetic analysis of PFV intasome integration with a supercoiled plasmid target DNA suggested that the reaction was complete by 5 min at 37 °C in the presence of a physiologically relevant salt concentration (29). To determine the kinetics of integration with nucleosome substrates, PFV intasomes were incubated with mononucleosomes reconstituted from recombinant human histone octamer and 601 NPS DNA labeled near one 5′ end with a Cy5 fluorophore (Fig. 2). Due to fluctuations of rotational or translational positioning, naturally occurring NPSs are not suitable substrates for precisely mapping integration sites at physiologically relevant ionic strength (20). Native PAGE revealed integration products that were shorter and longer than unreacted 601 NPS DNA. Products were readily apparent after 1 min incubation and plateaued at 5 min (Fig. 2). The kinetics of PFV intasome integration to a nucleosome target are consistent with integration to supercoiled plasmid substrate (29). These reaction products indicated several integration sites with 601 nucleosomes. However, the single-strand gap at the integration site junction alters the electrophoretic mobility of DNA and precludes precise determination of integration sites (32, 33).

Evaluation of integration products by native gel electrophoresis shows that the products are largely concerted integration. Half-site integration, where only one vDNA is joined to the target DNA, results in a branched DNA. The apparent mobility of a 147 bp NPS with a 40 bp vDNA branch is >250 bp (5). Half-site integration products were barely detectable with PFV intasome integration to nucleosomes (Fig. 2*A*). These products accounted for <1% of the fluorescent signal in any lane indicating that PFV intasomes primarily perform concerted integration to nucleosome targets.

### PFV integration into nucleosomes occurs in clusters

PFV intasome integration into nucleosomes was further analyzed by denaturing PAGE to precisely identify the strand breaks in nucleosome DNA. Each strand transfer introduces a single-strand break in the target DNA (Fig. 1). The 601 NPS DNA was fluorescently labeled near the 5′ end of either the top (T-Cy5 NPS) or bottom (B-Cy5 NPS) strand (Fig. 1, *B* and *C*). The NPS base pair that is 3′ to the strand scission, where the vDNA is joined, correlates to integration site position 0 (top strand) and position 3 (bottom strand). Denaturing electrophoresis allows visualization of the fluorescently labeled strand and calculation of its length. The lengths of the products indicate sites of integration that may be mapped to the 601 nucleosome crystal structure (31).

**Figure 3:**
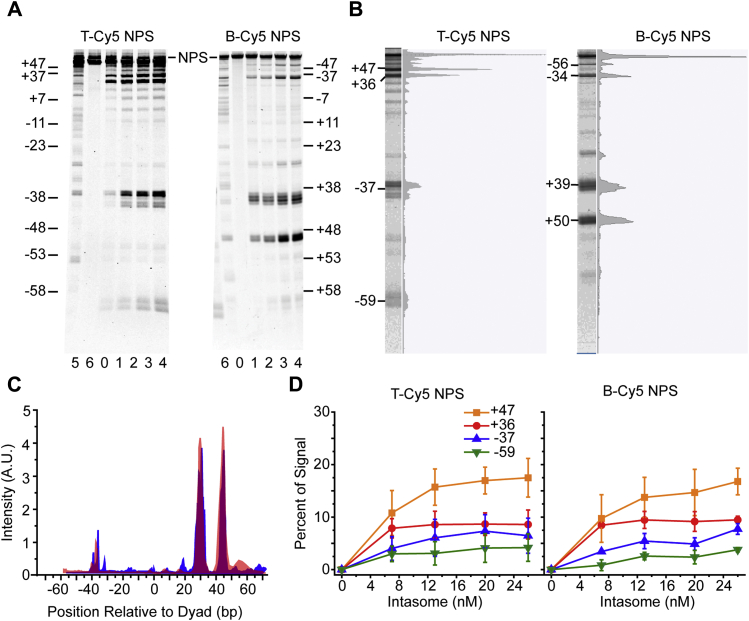
**PFV integration into 601 nucleosomes.***A*, denaturing PAGE analysis of PFV integration into 601 nucleosomes with a 5′ Cy5 label on the top strand (*left*, T-Cy5 NPS) or bottom strand (*right*, B-Cy5 NPS). The DNA size markers are expressed as the nucleosome positions relative to the dyad (–58 to +47 and +58 to –47, respectively). Lane 0 is 601 nucleosome substrate only (NPS). Lanes 1–4 include 7, 13, 20, and 26 nM PFV intasome. Lane 5 is naked 601 NPS DNA only. Lane 6 is naked 601 NPS DNA with 26 nM PFV intasome. *B*, signal density plots of lane 4 from T-Cy5 NPS (*left*) and B-Cy5 NPS (*right*) substrates were generated to quantitate integration activity at integration clusters. *C*, density plots of lane 4 from T-Cy5 NPS (*red*) and B-Cy5 NPS (*blue*) substrates were adjusted to a linear scale and overlaid. The B-Cy5 NPS density plot was slightly shifted to the left to account for the 4 bp between the points of joining. *D*, integration activity at the four major observed integration clusters over a gradient of PFV intasomes. T-Cy5 NPS (*left*) and B-Cy5 NPS (*right*) nucleosome substrates display similar profiles. Error bars indicate the standard deviation between at least three experiments with at least two independent PFV intasome and nucleosome preparations.

Increasing concentrations of PFV intasomes were added to nucleosomes with 601 NPS DNA labeled on either the top or bottom strand (Fig. 3*A*). Denaturing PAGE revealed that PFV integration occurred in small clusters, as previously reported (20). Four clusters of integration sites, each representing ≥2% of the fluorescent signal at the highest concentration of intasome, were observed. Data from lanes with the highest intasome concentration were transformed to signal density plots (Fig. 3*B*). These densitometric curves show each band as a peak of fluorescence. The four clusters of integration with T-Cy5 nucleosomes are at 601 NPS sites –59, –37, +36, and +47; the four B-Cy5 integration sites are at –56, –34, +39, and +50. The numbering for concerted integration products observed with T-Cy5 and B-Cy5 NPS differs by the 4 bp separation of strand transfer sites (Fig. 1*A*). For example, a T-Cy5 NPS band indicating a nick 5′ of -36 will correlate with a B-Cy5 NPS nick 5′ of -33. For simplicity, the clusters are hereafter referred to by the major observed integration position 0. As seen with other retroviral integration assays with nucleosomes, PFV integration is disfavored at the dyad region (17, 18).

The T-Cy5 NPS and B-Cy5 NPS density plots were converted from logarithmic to linear scales, overlaid, and shifted to account for the 4 bp difference between strand transfer events on the two strands (Fig. 3*C*). The overlay of linear-scale electropherograms shows identical integration profiles. Taken together, the identical T-Cy5 and B-Cy5 products in denaturing PAGE and the low frequency of >250 bp products observed in native PAGE confirm that PFV intasome integration to nucleosomes is concerted.

Integration efficiency for each cluster was derived from the area under the curve of each peak in the density plots (Fig. 3*D*). Following least square filtering to remove background signal, the sum of areas under the curve for all fluorescence peaks was determined, including unreacted substrate. The area under the curve for each individual peak was divided by the total fluorescence value to obtain the integration efficiency expressed as percent of signal. The higher-resolution PAGE regions revealed clusters of two to five bands: two bands at –59, five bands at –37, four bands at +36, and two bands at +47 (Fig. 3*A*). Quantitative analysis revealed a hierarchy of PFV integration preference where the –59 cluster was the least favored and the +47 cluster was the most favored (Fig. 3*D*). This hierarchy of site preference was the same for both T-Cy5 NPS and B-Cy5 NPS substrates. Mapping the integration clusters to a 601 nucleosome structure (PDB: 3LZ0) revealed that all sites are on exposed DNA helices and are not occluded by the histone octamer or adjacent DNA gyre (31).

### Integration activity of truncation mutants of PFV IN at physiological ionic strength

All domains of the inner PFV IN protomers have been structurally visualized and contact the viral DNA (2). When bound to a nucleosome, the CTD of one inner PFV IN protomer also contacts the amino terminal tail of H2A (5). In contrast, only the CCDs of the outer PFV IN protomers were resolved. The outer PFV IN CCDs appear structurally important for tetramer formation but do not contact viral or target DNAs (4, 5). Point mutations direct PFV IN(K120E) to the inner protomers and PFV IN(D273K) to the outer protomers (5). This strategy allows analysis of directed truncation mutations at the outer protomers with FL catalytically active inner protomers.

**Figure 4:**
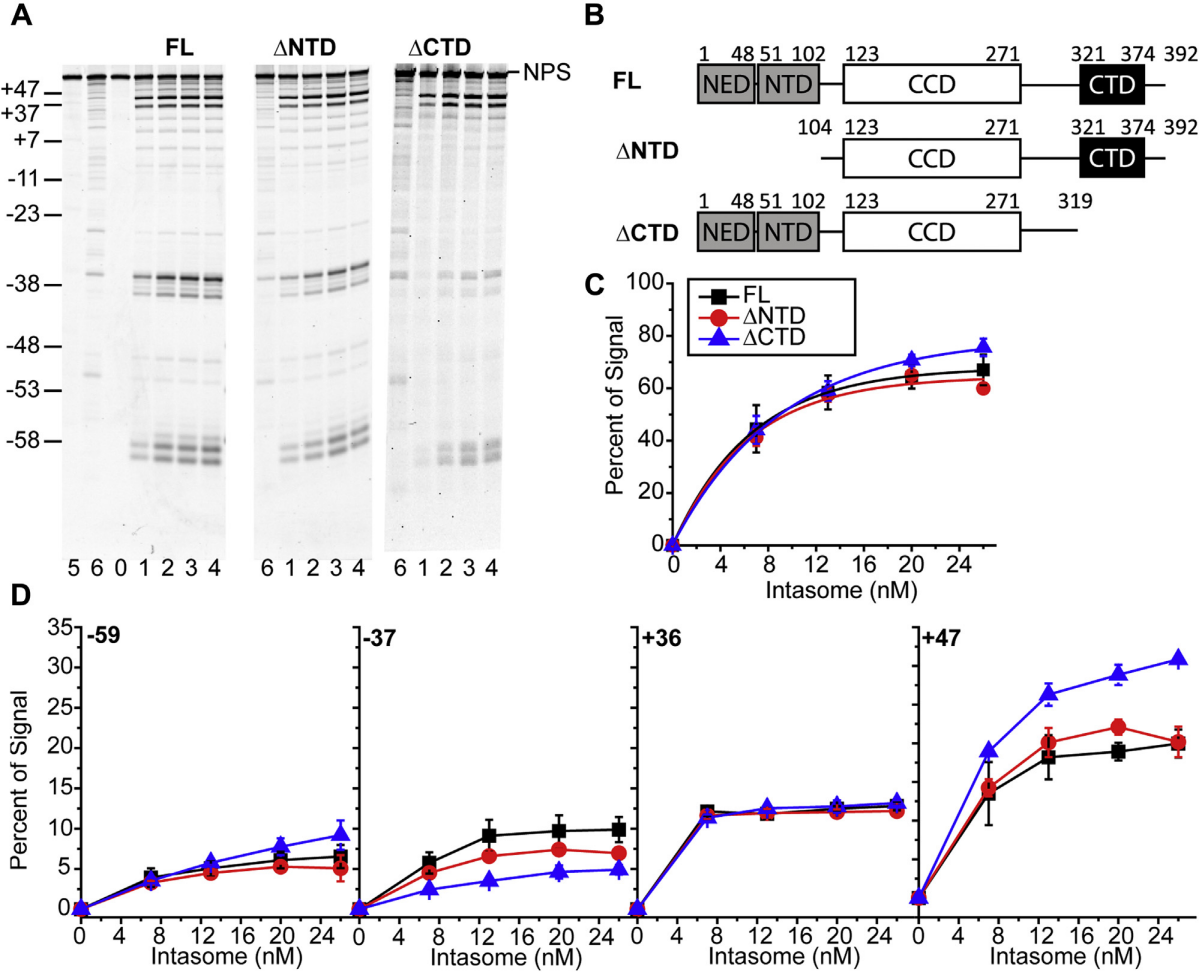
**Truncations of the outer PFV IN domains alter integration at 601 NPS +47**. *A*, PFV intasomes were generated with full-length (FL) PFV IN(K120E) at the inner subunits and truncations of PFV IN(D273K) at the outer subunits. FL PFV intasomes, intasomes with truncations of the amino terminal domains (ΔNTD), or intasomes with truncation of the carboxyl terminal domain (ΔCTD) were added to T-Cy5 601 nucleosomes. Reaction products were analyzed by denaturing PAGE. Lane 0 is 601 nucleosome substrate only (NPS). Lanes 1–4 include 7, 13, 20, and 26 nM PFV intasome. Lane 5 is naked 601 NPS DNA only. Lane 6 is naked 601 NPS DNA with 26 nM PFV intasome. The DNA size marker on the left is expressed as the nucleosome positions relative to the dyad. *B*, cartoons of the FL PFV IN domains and truncation mutants. *C*, the total integration to the 601 nucleosomes was calculated as the percentage of fluorescent signal in each lane below the unreacted target. *D*, integration efficiencies at each cluster –59, –37, +36, and +47 were calculated as fraction of the fluorescent signal in each lane. Error bars indicate the standard deviation between at least three experiments with at least two independent PFV intasome and nucleosome preparations.

We generated PFV intasomes with truncations of the outer protomers at the amino (ΔNTD) or carboxyl (ΔCTD) terminus (Fig. 4*B*). The PFV IN(D273K) truncation mutants were assembled with FL inner PFV IN(K120E). Increasing concentrations of these intasomes were added to T-Cy5 NPS nucleosomes and analyzed by denaturing PAGE to determine the effects of these outer protomer truncation mutants on PFV integration efficiency (Fig. 4*A*). The total integration efficiency was measured as the percentage of fluorescent products with faster mobility than unreacted NPS DNA in each lane. The FL and PFV IN(ΔNTD) intasome total integration efficiencies were not significantly different (67.2 ± 5.8% and 60.3 ± 2.1% total integration efficiency at 26 nM intasome, respectively, *p* > 0.05; Fig. 4*C*). At the highest concentration of intasomes, PFV IN(ΔCTD) intasomes (75.6 ± 3.3% total integration at 26 nM intasome) displayed slightly higher integration efficiency compared with FL intasomes (*p* > 0.05). These results suggest that the NTDs and CTDs of the outer PFV intasome protomers have little influence on the total integration efficiency with 601 nucleosome substrates.

PFV IN outer protomer truncation mutants were also evaluated for their integration site preference (Fig. 4*D*). Integration efficiency at each integration cluster was measured as the cluster's area under the curve as a percentage of the total fluorescence in the lane. Comparison of integration clusters indicates there is no significant difference between FL and PFV IN(ΔNTD) intasome integration efficiency (Fig. 4*D*, *p* > 0.05). FL and PFV IN(ΔCTD) integration efficiencies were not different at the –59 or +36 clusters. However, the integration efficiency of PFV IN(ΔCTD) intasomes significantly increased at the +47 cluster compared with FL intasomes (30.8 ± 0.3% and 20.0 ± 1.8% at 26 nM intasome, respectively, *p* < 0.01). Minor effects were also seen at the –37 cluster (9.9 ± 1.6% FL, 4.9 ± 0.6% ΔCTD, at 26 nM, *p* < 0.01). These results suggest that the outer PFV IN CTDs affect integration site preference, particularly at the 601 NPS +47 cluster. Thus the presence of the outer PFV IN CTDs seems to disfavor integration at NPS +47.

### Binding of PFV IN truncation mutants to nucleosomes at physiological ionic strength

**Figure 5:**
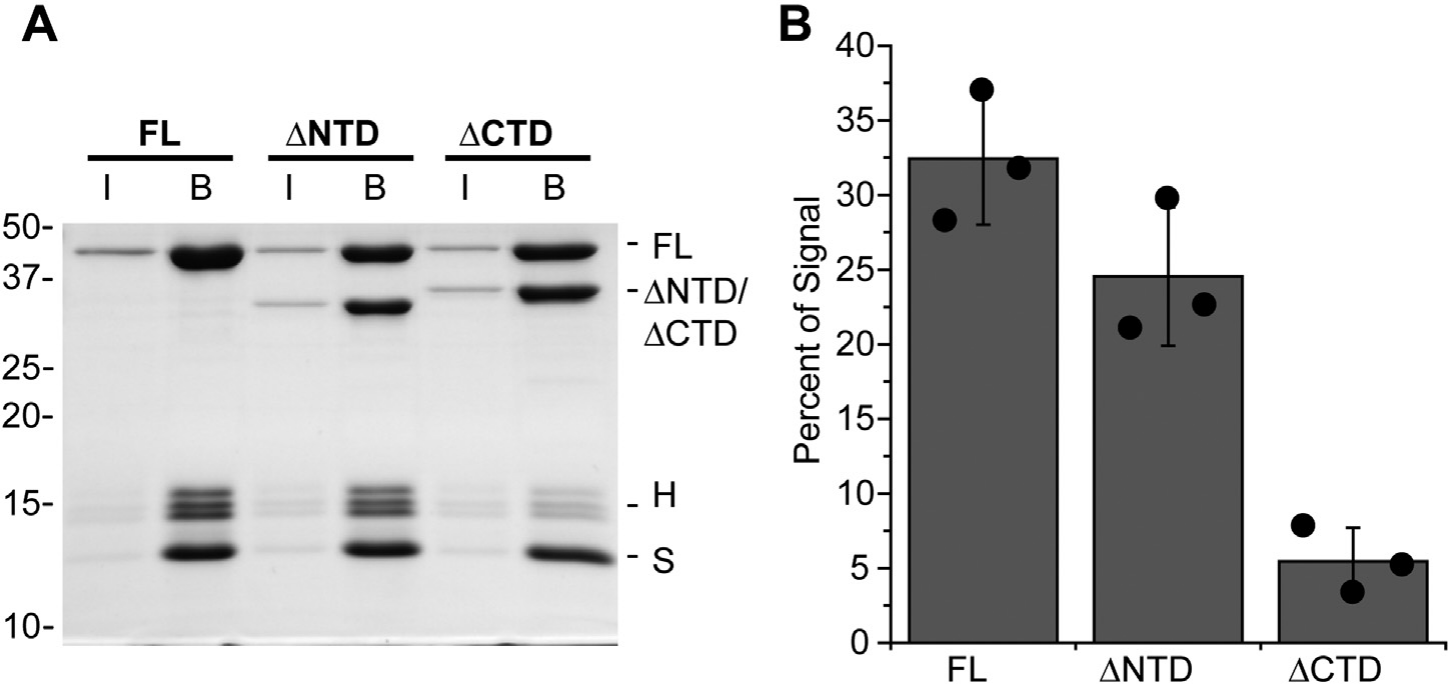
**Affinity of PFV IN truncation mutants for 601 nucleosomes in the presence of physiologically relevant monovalent salt concentration.***A*, FL, PFV IN(ΔNTD), and PFV IN(ΔCTD) intasomes were assembled with vDNA labeled with biotin. The intasomes were added to 601 nucleosomes and streptavidin-conjugated beads in the presence of 110 mM NaCl. The beads were extensively washed, analyzed by PAGE, and stained with Coomassie brilliant blue. Lane I, 5% of the total protein. Lane B, proteins associated with beads. Histones H3, H2B, and H2A (H). Streptavidin (S) and histone H4 have the same mobility. *B*, the total Coomassie signal in each lane was calculated, excluding the band of streptavidin and H4. The percentage of the total H3, H2B, and H2A signal in each lane B was calculated. *Black circles* indicate values from each experiment. Error bars indicate the standard deviation between three experiments with at least two independent PFV intasome and nucleosome preparations.

A previous study showed that biotin-conjugated PFV IN(ΔNTD) intasomes, but not PFV IN(ΔCTD) intasomes, were able to affinity precipitate nucleosomes from HeLa cells with streptavidin conjugated beads in the presence of 240 mM NaCl (5). The ability of PFV intasomes with outer protomer truncations to bind 601 NPS nucleosomes was similarly tested, except in the presence of physiologically relevant ionic strength conditions (Fig. 5). PFV intasomes were assembled with biotin-conjugated vDNA. Purified intasomes were added to 601 nucleosomes and streptavidin-conjugated beads for affinity precipitation. Proteins associated with the beads were analyzed by SDS-PAGE stained with Coomassie blue (Fig. 5*A*). In the presence of physiologically relevant ionic strength, the apparent binding of PFV IN(ΔNTD) intasomes to 601 nucleosomes was not significantly different from FL intasomes (Fig. 5*B*, *p* > 0.5). The interaction of PFV IN(ΔCTD) intasomes with nucleosomes was significantly reduced compared with FL intasomes (*p* = 0.01). The decreased binding of PFV IN(ΔCTD) intasomes to nucleosomes is in contrast to the slightly stimulating effect of PFV IN(ΔCTD) on total integration efficiency (Fig. 4*C*).

### PFV intasome integration to nucleosomes decreases with increasing ionic strength

**Figure 6:**
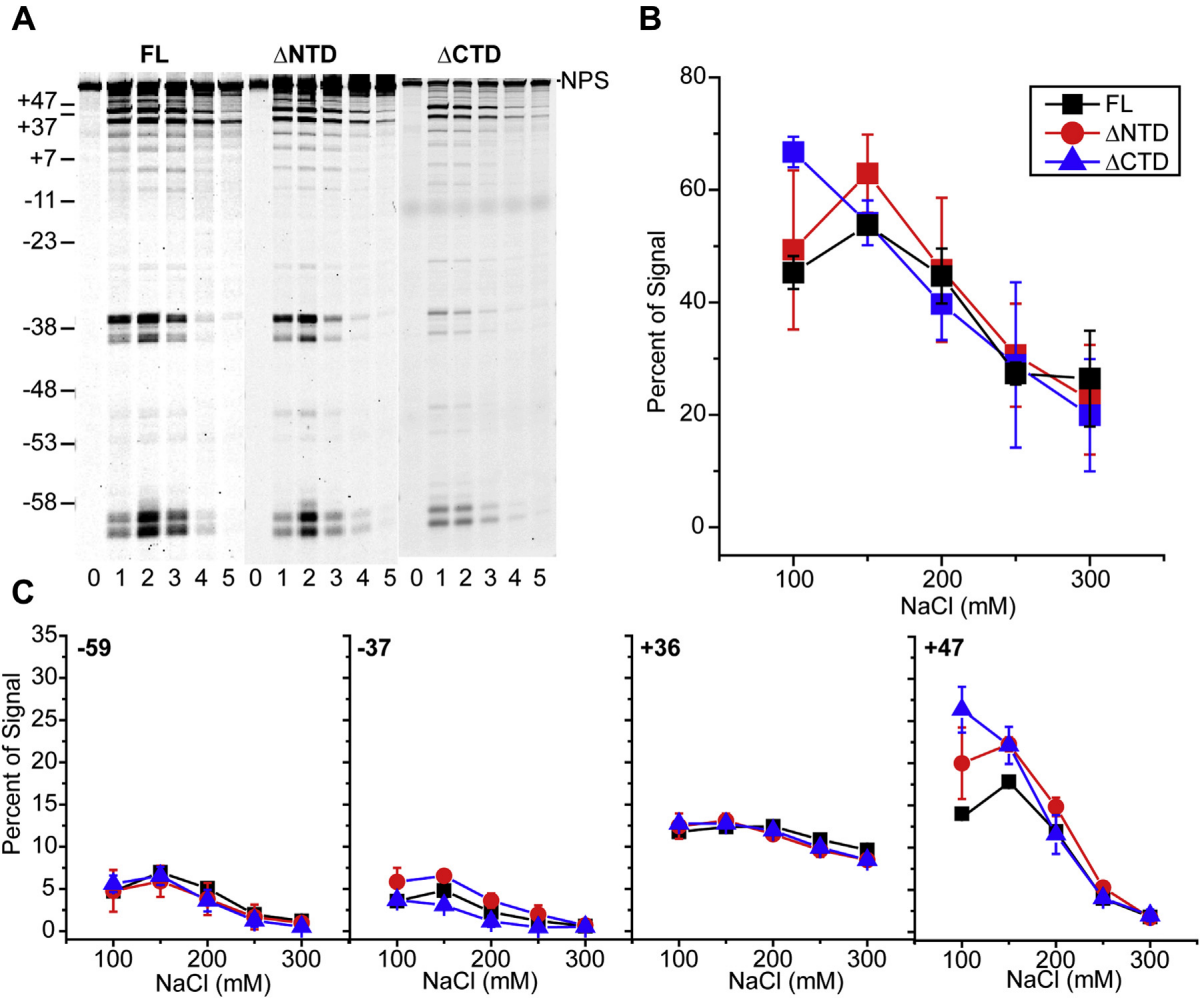
**Increasing salt concentration decreases PFV integration into nucleosomes.***A*, FL, PFV IN(ΔNTD), and PFV IN(ΔCTD) intasomes were added to T-Cy5 nucleosomes in the presence of increasing concentrations of NaCl. Lane 0 is 601 nucleosome substrate only (NPS). Lanes 1–5 include 26 nM PFV intasome and 100, 150, 200, 250, and 300 mM NaCl, respectively. The DNA size marker on the left is expressed as the nucleosome positions relative to the dyad. *B*, the total integration activity was calculated as the percentage of fluorescent signal below the NPS DNA band. *C*, integration efficiencies at each cluster –59, –37, +36, and +47 were calculated as the percentage of the fluorescent signal in each lane. Error bars indicate the standard deviation between at least three experiments with at least two independent PFV intasome and nucleosome preparations.

To further explore the role of ionic strength on PFV intasome interactions with nucleosomes, integration was assayed in the presence of increasing NaCl concentration (Fig. 6). All PFV intasomes displayed a decrease of total integration efficiency at >200 mM NaCl. The highest integration activities were seen in the physiologically relevant concentration range of 100–150 mM NaCl (Fig. 6*B*).

The possibility that decreased integration activity at >200 mM NaCl was due to instability of the nucleosomes was tested. Nucleosome stability was measured by Förster resonance energy transfer (FRET) between a Cy3 fluorophore at H2A(K119C) and a Cy5 moiety at the entry–exit region of the 601 NPS 4 bp from one end. FRET analysis showed the 601 nucleosomes are stable in the presence of 110 mM and 300 mM NaCl, but not 600 mM NaCl (Fig. S1). The FRET was not significantly different at 110 and 300 mM NaCl (*p* > 0.05) indicating that the nucleosomes are stably positioned. The decrease of integration efficiency at >200 mM NaCl was not due to nucleosome instability.

Decreased integration efficiencies could be due to reduced PFV IN enzymatic activity in the presence of relatively high NaCl concentrations. To evaluate enzymatic activity, FL PFV intasomes were added to a supercoiled plasmid target DNA in the presence of increasing NaCl concentration (Fig. S2). PFV intasomes displayed the highest activity with supercoiled plasmid in 150–200 mM NaCl. Integration activity decreased at concentrations >200 mM NaCl, similar to the decrease observed with nucleosomes. Thus the decrease of integration efficiency with nucleosome targets between 200 and 300 mM NaCl may be due to a decrease of intasome enzymatic activity. Interestingly, PFV intasomes retain enzymatic activity at the relatively high 300 mM NaCl concentration. At ≤200 mM NaCl, the trends of FL PFV intasome integration efficiencies to nucleosome or supercoiled plasmid targets are dissimilar; FL PFV intasome integration to nucleosomes peaks at 150 mM NaCl, but peaks at 200 mM NaCl with supercoiled plasmid.

There were no significant differences between the total integration efficiencies of all intasomes at ≥150 mM NaCl (Fig. 6*B*, *p* > 0.05). However, there were significant differences in integration efficiency at 100 mM NaCl. At this concentration, PFV IN(ΔCTD) intasomes (66.7 ± 2.7%) displayed higher total integration efficiency compared with FL PFV intasomes (45.3 ± 2.9%, *p* < 0.01) and PFV IN(ΔNTD) intasomes (48.1 ± 14.2%, *p* > 0.05). Examination of integration site clusters revealed that the differences at 100 mM NaCl are largely due to integration at NPS position +47 (Fig. 6*C*). Integration at –59 and +36 showed no differences between the intasomes at any of the NaCl concentrations (*p* > 0.05). There was little reduction of integration at +36 between 100 and 300 mM NaCl (<5% difference) with any of the intasomes, suggesting that integration at this site is particularly unaffected by changes in ionic strength or the presence of outer PFV IN protomer domains. Integration at +47 showed significant differences only at 100 mM NaCl. PFV IN(ΔCTD) intasome integration (26.0 ± 2.7%) at +47 was significantly greater than FL PFV intasomes (12.0 ± 0.6%, *p* < 0.01). PFV IN(ΔNTD) intasome integration at +47 (20.0 ± 4.2%) was also significantly more than FL PFV intasomes (*p* = 0.03). Together this data suggests that while integration by all the intasomes is readily detected in the presence of nonphysiological high ionic strength, differences in integration of truncation mutants are only apparent in physiological ionic conditions.

### PFV intasome binding to nucleosomes decreases at a nonphysiologically high salt concentration

**Figure 7:**
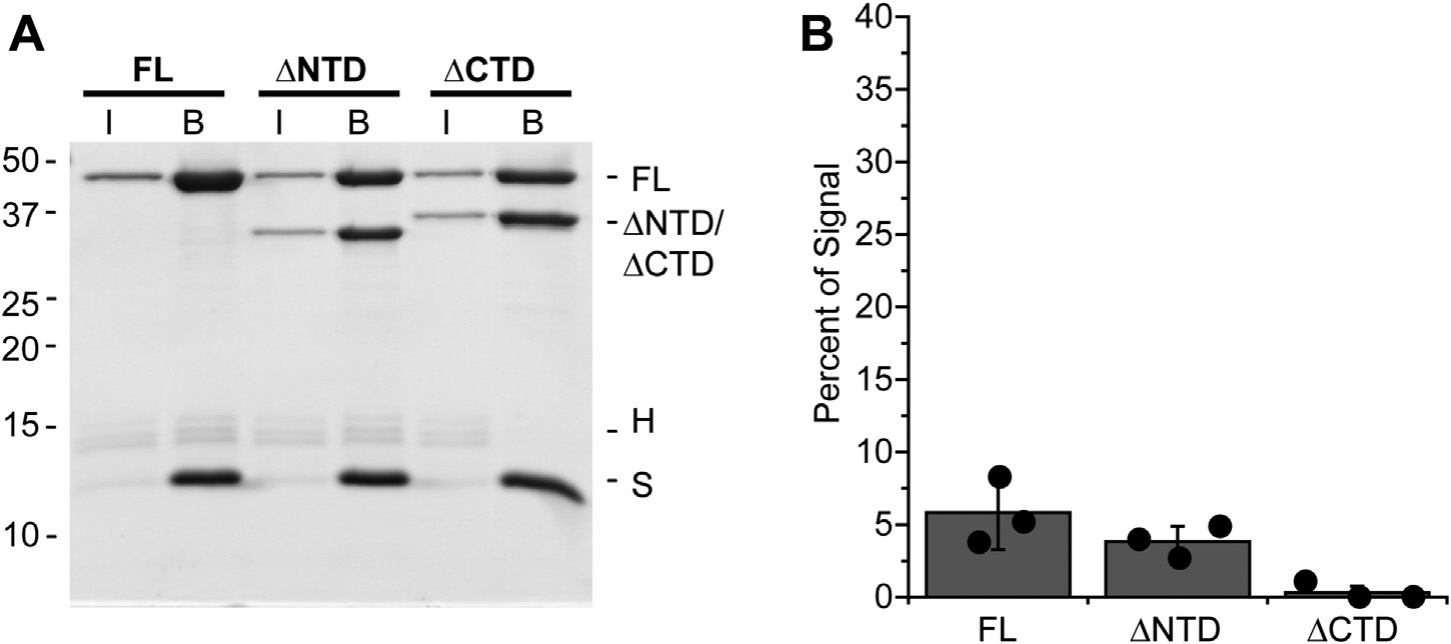
**Affinity of PFV IN truncation mutants for 601 nucleosomes in the presence of a relatively high salt concentration.***A*, FL, PFV IN(ΔNTD), and PFV IN(ΔCTD) intasomes with biotinylated vDNA were added to 601 nucleosomes and streptavidin-conjugated beads in the presence of 300 mM NaCl. The beads were extensively washed, analyzed by PAGE, and stained with Coomassie brilliant blue. Lane I, 5% of the total proteins. Lane B, proteins associated with beads. Histones H3, H2B, and H2A (H). Streptavidin (S) and histone H4 have the same mobility. *B*, The total Coomassie signal in each lane was calculated, excluding the band of streptavidin and H4. The fraction of the total signal in each lane B associated with the H3, H2B, and H2A bands was calculated. *Black circles* indicate values from each experiment. Error bars indicate the standard deviation between three experiments with at least two independent PFV intasome and nucleosome preparations.

Truncation mutant intasomes displayed integration efficiencies equivalent to FL PFV intasomes in the presence of 300 mM NaCl. We tested the effect of 300 mM NaCl on binding of intasomes to 601 nucleosomes (Fig. 7). Intasomes with biotinylated vDNA were incubated with 601 nucleosomes and streptavidin-coated beads. Proteins bound to the beads were visualized by SDS-PAGE. All intasomes displayed reduced interactions with nucleosomes in 300 mM NaCl compared with 110 mM NaCl (Fig. 7 compared with Fig. 5). FL PFV intasome binding to nucleosomes in 300 mM NaCl (5.9 ± 3.4%) displayed approximately eightfold reduction compared with binding at 110 mM NaCl (45.2 ± 10.1%, Fig. 5). In the presence of 300 mM NaCl, PFV IN(ΔNTD) intasome binding to nucleosomes was similar to FL intasomes (4.3 ± 1.4%, *p* > 0.5). There were no detectable stable interactions between PFV IN(ΔCTD) intasomes and 601 nucleosomes at the higher salt concentration. At both physiologically relevant 110 mM NaCl and nonphysiological 300 mM NaCl, the binding of PFV IN(ΔCTD) intasomes with 601 nucleosomes was significantly less than FL intasomes. This data is in agreement with a previous report evaluating FL and PFV IN(ΔCTD) intasome binding to nucleosomes in the presence of 240 mM NaCl (5). Despite the difference in binding, the FL and PFV IN(ΔCTD) intasomes displayed equal integration efficiencies to 601 nucleosomes at 300 mM NaCl (Fig. 6*B*). In addition, PFV IN(ΔCTD) intasomes have reduced binding to 601 nucleosomes in 100 mM NaCl, yet display greater integration efficiency at this salt concentration (Fig. 5*B*). The extent of stable binding of FL and PFV IN(ΔCTD) intasomes to nucleosomes does not correlate with the integration efficiencies.

### Effects of histone tails on PFV intasome integration and binding at physiological ionic strength

**Figure 8:**
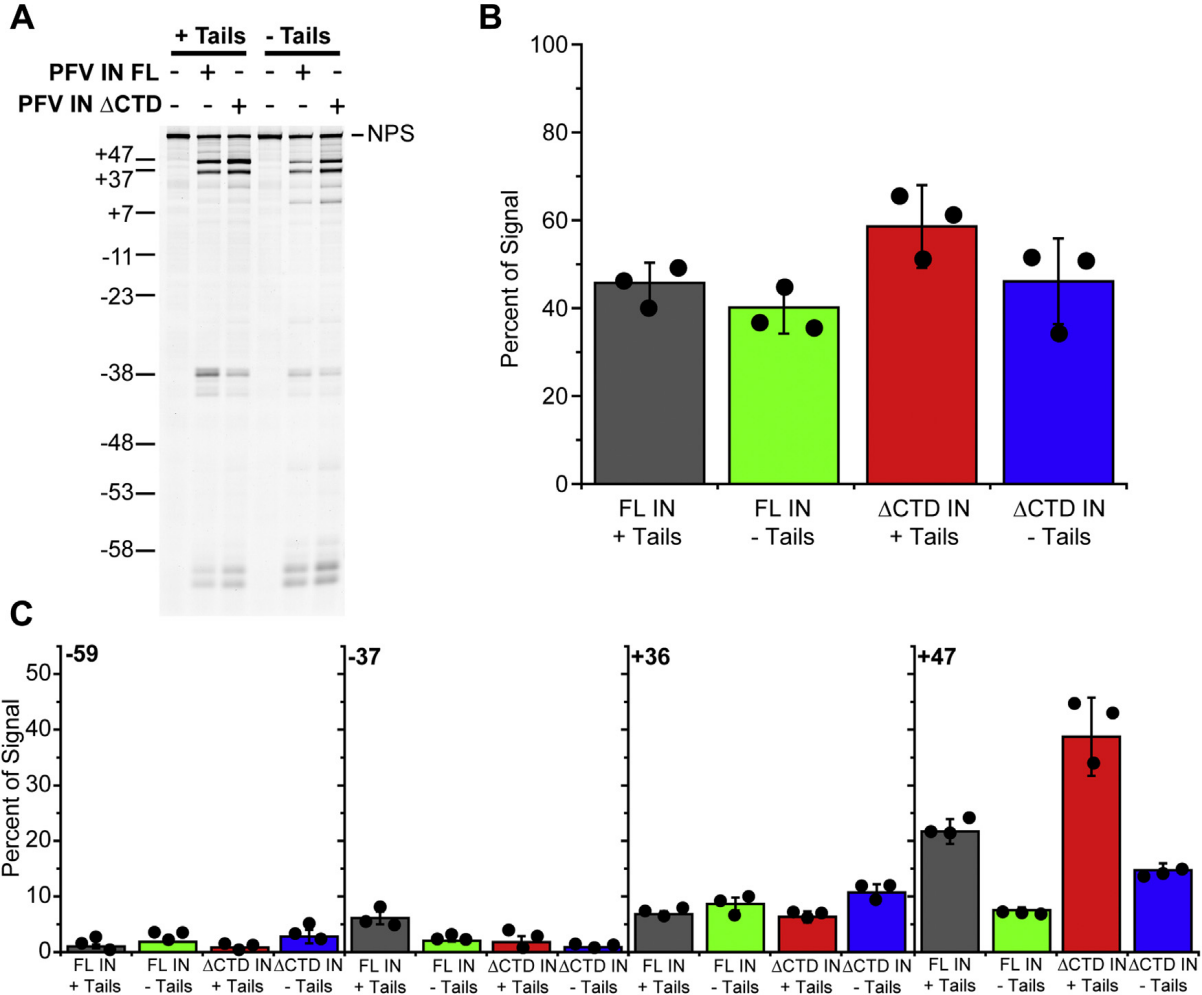
**FL PFV and PFV IN(ΔCTD) intasome integration to trypsinized nucleosomes.***A*, 601 nucleosomes were treated with trypsin to remove the histone tails (–Tails). FL or PFV IN(ΔCTD) intasomes were added to +Tails or –Tails nucleosomes in the presence of 110 mM NaCl. Integration products were analyzed by denaturing PAGE. The DNA size marker on the left is expressed as the nucleosome positions relative to the dyad. *B*, the total integration of each intasome to the 601 nucleosomes was calculated as the percentage of fluorescent signal in each lane below the unreacted target. *C*, integration efficiencies at each cluster –59, –37, +36, and +47 were calculated as the percentage of the fluorescent signal in each lane. *Black circles* indicate values from each experiment. Error bars indicate the standard deviation between at least three experiments with at least two independent PFV intasome and nucleosome preparations.

The binding of PFV intasomes to nucleosomes may be mediated in part by an interaction between the CTD of an inner PFV IN protomer and the amino terminal tail of one H2A (5). It is unclear if the outer PFV IN protomer CTDs or NTDs interact with the second H2A tail of a nucleosome or any other histone tail. Limited trypsin digestion of assembled nucleosomes removes the histone tails while leaving the core histone octamer and NPS intact (34, 35). Recombinant 601 nucleosomes were treated with trypsin to remove the histone tails (Fig. S3). We assayed the integration efficiencies of FL and PFV IN(ΔCTD) intasomes with trypsinized (–Tails) 601 nucleosomes in conditions of physiological ionic strength (Fig. 8). Slight variations in total integration efficiencies to +Tails or –Tails nucleosomes were observed for FL or PFV IN(ΔCTD) intasomes, but these were not significant (Fig. 8*B*, *p* > 0.05).

Although the variations in total integration efficiencies were modest with –Tails nucleosomes, integration at NPS +47 displayed significant differences (Fig. 8*C*). Trypsinization led to a decrease of FL PFV intasome integration at +47 (21.7 ± 2.2% with +Tails histones, 6.2 ± 1.9% with –Tails histones, *p* < 0.01). Similarly, trypsinization led to a decrease of PFV IN(ΔCTD) intasome integration at +47 (44.4 ± 6.5% with +Tails histones, 14.7 ± 1.2% with –Tails histones, *p* < 0.01). There was no significant difference between any of the conditions at –59 or +36 (*p* > 0.05). Slight reductions (<5% differences) of integration efficiency to NPS –37 were observed with truncation of IN or removal of the histone tails. These data indicate that the CTDs of the outer PFV subunits and histone tails primarily affect integration at the +47 site of 601 nucleosomes. Deletion of the outer PFV IN CTDs increases integration at +47, while deletion of the histone tails decreases integration at this site. This suggests that the outer PFV IN CTDs prevent integration at +47 while the histone tails enhance integration at +47 (Figs. 4 and 8)

**Figure 9:**
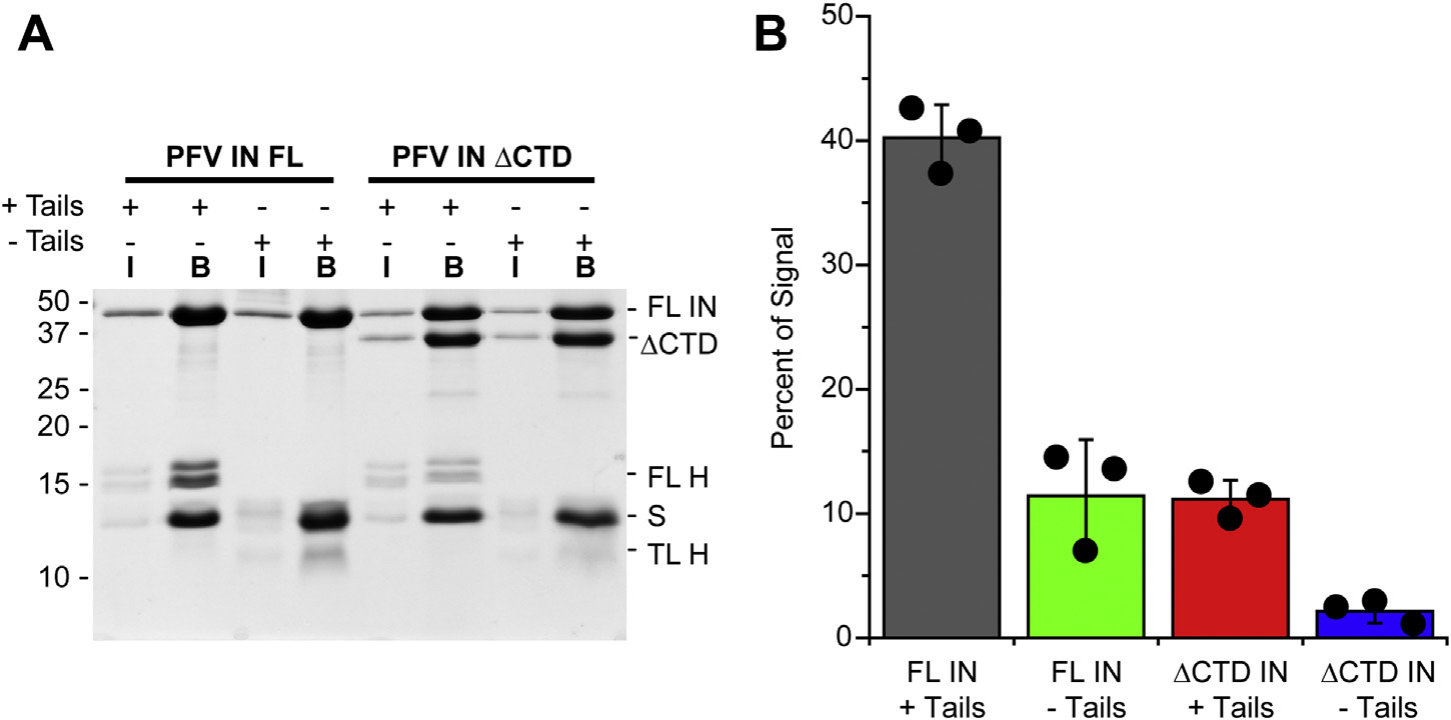
**Affinity of FL PFV and PFV IN(ΔCTD) intasomes for trypsinized 601 nucleosomes.***A*, 601 nucleosomes were treated with trypsin to remove the histone tails (–Tails). Biotinylated FL PFV and PFV IN(ΔCTD) intasomes were added to +Tails or –Tails 601 nucleosomes and streptavidin-conjugated beads in the presence of 110 mM NaCl. The beads were extensively washed, analyzed by PAGE, and stained with Coomassie brilliant blue. Lane I, 5% of the total protein. Lane B, proteins associated with beads. FL histones H3, H2B, and H2A (H). Streptavidin (S) and histone H4 have the same mobility. TL H3, H2A, and H2B have a similar mobility to streptavidin. *B*, the total Coomassie signal in each lane was calculated, excluding streptavidin. The percentage of the total signal in each lane B associated with the FL H3, H2B, and H2A bands or TL H4 was calculated. *Black circles* indicate values from each experiment. Error bars indicate the standard deviation between three experiments with at least two independent PFV intasome and nucleosome preparations.

Binding of PFV intasomes to –Tails nucleosomes was evaluated in physiological ionic strength conditions (Fig. 9). Biotinylated intasomes were added to +Tails or –Tails nucleosomes and streptavidin-conjugated beads. Proteins bound to the beads were analyzed by SDS-PAGE. The most binding was observed with FL PFV intasomes and +Tails 601 nucleosomes (40.2 ± 2.7%). Removal of the histone tails by trypsin significantly reduced FL PFV intasome binding (11.2 ± 1.5%, *p* < 0.01). This reduction is predicted if a PFV IN inner CTD binds to an H2A amino terminal tail. The deletion of the outer PFV IN CTDs leads to a reduction of binding with +Tails nucleosomes suggesting that these IN domains participate in nucleosome binding (Figs. 5 and 9). The interactions of PFV IN(ΔCTD) intasomes with –Tails nucleosomes were reduced, but detectable (Fig. 9, 2.5 ± 0.95%).

The significant difference between binding to –Tails nucleosomes by FL PFV intasomes (11.2 ± 1.5%) and PFV IN(ΔCTD) intasomes (2.5 ± 0.95%, *p* = 0.03) suggests that these outer domains may have alternative binding sites other than histone tails. The outer protomer PFV IN CTD may be binding to a different histone protein region or the NPS DNA. Interestingly, these significant differences in binding do not correlate with the integration efficiencies. The binding of intasomes to nucleosomes does not predict integration efficiency.

## Discussion

Previous single-molecule studies of PFV intasomes revealed that binding to target DNA is inherently distinguishable from integration catalysis (24). PFV intasomes are able to bind naked linear DNA and diffuse in continuous contact with the DNA, searching 1.6 kb in 2.1 s. In that report 800 individual DNA-binding events were observed at a physiologically relevant salt concentration, but only three integration events were seen. These observations indicated that PFV intasomes are able to search DNA without completing the integration reaction.

Here we have shown that PFV intasome binding to nucleosomes is also distinct from integration. Deletion of the PFV intasome outer protomer CTDs dramatically reduced binding to nucleosomes under conditions of physiologic ionic strength. However, the integration activity of PFV IN(ΔCTD) intasomes was slightly increased compared with FL PFV intasomes. The greater binding of FL PFV intasomes to nucleosomes without a concomitant increase of integration efficiency suggests that most nucleosome-binding events are unproductive or binding in a catalytically incompetent conformation. Similarly, FL PFV intasome or PFV IN(ΔCTD) intasome binding to trypsinized nucleosomes is significantly less than FL nucleosomes, yet the total integration efficiencies are not significantly different at a physiologically relevant salt concentration. This data supports the concept that the initial binding step is not rate determining for catalysis.

The total integration efficiency of FL PFV intasomes and truncation mutants was identical in the presence of salt concentrations higher than physiological. There was also no difference in site preference at high ionic strengths. While nonphysiological ionic conditions may be appropriate for structural analysis, previous studies have shown the importance of assays for enzymatic or dynamic activities in physiological conditions (36, 37, 38). For example, DNA mismatch repair proteins are only able to discriminate mismatched DNA from homologous DNA at a physiologically relevant salt concentration; outside the physiological window, mismatch repair proteins inappropriately recognize homologous DNA (36).

PFV intasome integration activity is sensitive to ionic strength. The lifetime of PFV intasome binding to naked DNA is inversely related to ionic strength (24). In the presence of 25 mM NaCl, PFV intasome binding to naked linear DNA is 29 ± 3.5 s, in 100 mM NaCl the lifetime is 2.1 ± 0.1 s, but in ≥150 mM NaCl binding is undetectable at 250 ms resolution (24). At concentrations >200 mM NaCl, PFV intasome integration to a supercoiled plasmid target decreases. Whether this is due to decreased catalytic activity or decreased binding to the target DNA, the result is fewer integration products. Interestingly, integration at NPS +36 was refractory to increasing NaCl concentrations. This site has been particularly useful for structural studies performed in the presence of relatively high ionic strength conditions (5, 39). Integration in physiologic ionic conditions is likely to favor more than a single site on nucleosomes (20).

Multiple studies have shown that retroviral integration preferentially occurs at distorted DNA regions of a nucleosome or bent DNA (17, 18, 40). Structural analysis of the 601 nucleosome reveals an extreme kink at ±46 (31, 41). The DNA path on the 601 nucleosome deviates from the DNA path of the α-satellite nucleosome structure at precisely this region suggesting that the NPS DNA structure is partially sequence-dependent (42, 43). The 601 NPS is significantly untwisted at this region and exhibits increased DNA backbone distortion. By employing an NPS with clearly defined DNA spatial characteristics, our data confirms and extends the model that retroviral integrases prefer the most distorted region of nucleosome-bound DNA.

Although PFV integration favored 601 nucleosome position +47, there was no preference for –47, which displays the same DNA distortion (31, 41). Single-molecule unzipping studies have demonstrated that histone octamer binding to each half of the 601 NPS DNA is not equivalent (44, 45). In these studies, force-induced unwrapping of the 5′-half (left half, negative numbering) of the 601 NPS DNA appears more flexible but tightly bound to the nucleosome core; the 3′-half (right half, positive numbering) of the 601 NPS DNA appears more rigid but less tightly bound. This is most likely because a lack of DNA flexibility decreases the ability of the histone octamer to form multiple contacts required for tight NPS binding. Structures of the PFV intasome bound to a nucleosome suggest that the NPS DNA is lifted from the histone octamer (5, 39). The tight binding of the left half of the 601 NPS to the histone octamer may prevent integration at –47 by precluding access to the DNA. This may also explain why there is less integration at –37 compared with +36.

Taken together these data indicate that PFV intasome binding to nucleosomes does not determine integration efficiency. Intasome binding to nucleosomes appears to be mediated by histone tails and PFV IN CTDs. One H2A tail has been shown to interact with the PFV IN CTD of an inner protomer (5). For trypsinized nucleosomes, it is unclear whether the CTDs of the outer protomers are binding the NPS DNA or histone protein. A previous study suggested that FL PFV intasomes and PFV IN(ΔCTD) intasomes bind supercoiled plasmid DNA equally (29). There is no structural information to indicate a binding partner for the outer PFV IN protomer CTDs. In spite of the effects on binding, the total integration efficiencies of PFV intasomes displayed minimal differences with deletion of outer subunit domains or histone tails under conditions of physiologically relevant ionic strength. The data suggests that most PFV intasome-binding events to nucleosomes are unproductive.

Other retroviruses employ a host integration cofactor that tethers the integration complex to chromatin (25, 26, 46, 47, 48, 49). For example, host protein LEDGF/p75 has an amino terminal PWWP domain reported to bind histone PTM H3K36me3 in actively transcribed genes and a carboxyl terminal integrase-binding domain (IBD) that binds to HIV-1 integration complexes (28). LEDGF/p75 tethers the HIV-1 complex to nucleosomes bearing H3K36me3 and directs integration to actively transcribed genes. Similarly, Brd4 is able to direct MLV integration near transcription start sites (25, 26). The ability of retroviral integrases to pirate a host chromatin-binding protein directs integration site selection and may also enhance their integration efficiency to chromatin (25, 26, 28). LEDGF/p75 has also been shown to enhance the stability of the HIV-1 intasome, which may serve as an additional mechanism to improve integration efficiency during infection (50). Future efforts may reveal whether host tethering factors increase integration efficiency *via* more productive binding of the integration complex to chromatin.

## Experimental procedures

### DNA substrates

A DNA oligonucleotide with an internal amino-modified thymine (T∗) at the fourth base from the 5′ end (5′-CTGT∗AGAATCCCGGTGCCGAGGCCGCT-3′ Integrated DNA Technologies) was labeled with Cy5-NHS ester (GE Healthcare). The labeled oligonucleotide was purified by reverse-phase HPLC with a Poroshell 120 EC-C18 column (Agilent Technologies). The 147 bp 601 NPS was amplified from pDrive-601 NPS with the Cy5 labeled oligonucleotide and DNA oligonucleotide 5′-ACAGGATGTATATATCTGACACGTGCCTGGA-3′. The resulting Cy5 labeled 601 NPS DNA was purified by ion-exchange HPLC with a Gen-Pak Fax column (Waters).

PFV vDNA substrates were annealed DNA oligonucleotides oKEY616 5′-ATTGTCATGGAATTTTGTATATTGAGTGGCGCCCGAACAG-3′ and oKEY675 5′-CTGTTCGGGCGCCACTCAATATACAAAATTCCATGACA-3′ (Integrated DNA Technologies). When vDNA was modified with Cy5 or biotin, the moiety was at the 5′ end of oKEY675.

### Nucleosomes

Recombinant human histones H2A or H2A(K119C), H2B, H3, and H4 were expressed and purified as described (10). Purified histone H2A(K119C) was labeled with Cy3-maleimide (GE Healthcare). Cy3-H2A allowed visualization of fluorescent histone octamers by gel analysis and FRET confirmation of predicted Cy5-NPS positioning. Histone octamers were refolded at equimolar histone concentrations and purified by Superose 12 10/300 gel filtration chromatography (GE Healthcare or Lumiprobe). Nucleosomes were reconstituted with 147 bp 601 DNA and histone octamer by double dialysis. The products were separated by sucrose gradient velocity centrifugation. Gradient fractions were analyzed by native PAGE and imaged using a Typhoon 9410 variable mode fluorescent imager (GE Healthcare). Fractions with fluorescent NPS DNA bound by nucleosomes were combined, concentrated with Amicon Ultra centrifugal filters (EMD Millipore), and stored at 4 °C. All experiments were performed with at least two independent nucleosome preparations, derived from independent histone octamer refoldings. Nucleosomes were treated with trypsin (Sigma-Aldrich) at an enzyme-to-substrate ratio of 1:120 w/w for 3 h at ambient temperature. Digestion was terminated by the addition of tenfold molar excess soybean trypsin inhibitor (Sigma-Aldrich). To confirm the deletion of histone tails, nucleosomes were labeled on free amino groups by incubating with twofold molar excess Cy5 NHS ester (Lumiprobe) in 10 mM Tris-HCl (pH 7.5), 25 mM NaCl for 1 h at ambient temperature and analysed by PAGE.

The steady-state ensemble FRET measurements were carried out in a spectrofluorometer (FluoroMax-4, Horiba Scientific) in the presence of 110, 300, and 600 mM NaCl. In a quartz cuvette (15 μl volume, 1 cm path length), ∼10 nM nucleosomes modified with Cy3 on H2A and Cy5 at the forward entry–exit site were excited at 519 nm wavelength with 5 nm slit width. Direct Cy3 excitation results in Cy3 emission and concomitant Cy5 emission through FRET. The emission spectra were recorded from 550 nm to 720 nm with a 10 nm slit width and 1 nm wavelength resolution at 0.2 s data integration time. The raw spectra were then corrected for the wavelength-dependent quantum efficiency of the detector and lamp fluctuations. The intensity count under Cy3 (550–600 nm) peak and Cy5 (640–720 nm) peaks was determined by fitting each peak with a Gaussian distribution and integrating the area under the curve (OriginPro 9.1, OriginLab). The ratiometric FRET efficiencies were determined using these corrected intensities (I) as I_Cy5_/(I_Cy3_ + I_Cy5_). Measurements were performed at 37 °C in triplicate with three independent nucleosome preparations. The loss of signal due to photobleaching was minimized by degassing the imaging buffers for several hours before performing measurements.

### PFV integration

PFV intasomes were assembled and purified as previously described (51). All experiments were performed with at least two independent intasome purifications. PFV intasomes with truncation mutants at the outer IN positions were assembled using equimolar concentrations of PFV IN(K120E) and truncation mutants PFV IN(D273K, ΔNEDΔNTD) or PFV IN(D273K, ΔCTD) (5). Unless otherwise noted, integration reactions contained 10 mM Bis-tris propane-HCl (pH 7.5), 110 mM NaCl, 5 mM MgSO_4_, 4 μM ZnCl_2_, 10 mM DTT, the indicated concentration PFV intasomes, and 15 ng NPS DNA in a final volume of 15 μl. Time course reactions included 13 nM PFV intasomes. Reactions were incubated at 37 °C for 5 min and stopped with 0.1 volumes stop solution (5% SDS, 10 mg/ml proteinase K) and 0.05 volumes 500 mM EDTA. Reactions were further incubated at 55 °C for 1 h. Products were separated by native or denaturing PAGE and scanned with a Typhoon 9410 variable mode fluorescent imager (GE Healthcare) or Sapphire Biomolecular Imager (Azure Biosystems). Molecular weights were calculated by fitting standards (GeneScan 120 LIZ Size Standard, Thermo Fisher Scientific) to an exponential curve. The standard curve was then used to determine the molecular weight of each band (±3 nucleotides [nt]) depending on pixel position.

To calculate the relative intensity of each band or integration cluster, the total fluorescence intensity of each lane was quantified (BioNumerics 7.6, Applied Maths). This software converts each gel lane to a densitometric curve in which each band appears as a peak (Fig. 3*B*). Least square filtering was used to remove background. The total fluorescence intensity of each lane is the sum of all bands in a lane. The integrated area under each band peak was then calculated. This value was divided by the total fluorescence of the lane and multiplied by 100 to yield the percent of total fluorescent signal (OriginPro 9.1, OriginLab). The percentage of the total fluorescence in the lane is referred to as integration efficiency. Individual peaks in a cluster of bands could not be integrated individually as a result of overlapping pixel densities. The data are presented as averages ±standard deviation (sd) of at least three independent experiments. *p* values were determined using a two-tailed *t*-test at a 95% confidence interval. Total integration efficiency was determined by subtracting the fraction of unreacted NPS from the total fluorescent signal in each lane.

### PFV intasomes binding to nucleosomes

Binding to nucleosomes was assayed by affinity precipitation of biotinylated intasomes with streptavidin-coated beads. PFV intasomes were assembled with biotinylated vDNA. In total, 10 μg of PFV intasomes was added to 10 μg of 601 nucleosomes (EpiCypher) in wash buffer (50 mM HEPES (pH 7.5), 110 mM or 300 mM NaCl, 10% glycerol, 1 mM DTT, 0.1% Tween, and 1 μg/ml BSA) in a final volume of 350 μl. Samples were incubated on ice for 20 min followed by room temperature for 30 min. In total, 70 μl of streptavidin-conjugated magnetic beads (Dynabeads M-280 streptavidin, Invitrogen) was washed with three volumes of wash buffer and resuspended in 17.5 μl of wash buffer. The magnetic beads were added to 333.5 μl of each sample. The remaining 17.5 μl of intasomes with nucleosomes was saved for gel analysis as a 5% input control. Samples with magnetic beads were slowly rotated at room temperature for 1 h. The beads were then washed with three volumes of wash buffer. Beads were resuspended in phosphate-buffered saline (PBS, Sigma-Aldrich) and SDS-PAGE loading dye, boiled for 10 min, and analyzed by SDS-PAGE. Gels were stained with Coomassie Brilliant Blue R-250 (Amresco) and imaged (Epson scanner or Azure Sapphire Biomolecular Imager). Coomassie stained protein bands were quantitated (ImageJ). The total signal in each lane was determined, excluding the overlapping bands of streptavidin and H4. The histone bands were calculated as the percentage of the total signal in each lane. Averages and standard deviations were derived from at least three experiments with two independent PFV intasome and nucleosome preparations.

## Data availability

All data supporting the findings of this study are available within the article and supporting information.

## Supporting information

This article contains supporting information.

## Conflict of interest

The authors declare that they have no conflicts of interest with the contents of this article.
