# Supplementary material for: Retroviral prototype foamy virus intasome binding to a nucleosome target does not determine integration efficiency
Source: J Biol Chem. 2021 Mar 18;296:100550. doi: 10.1016/j.jbc.2021.100550 (PMC8050864; doi:10.1016/j.jbc.2021.100550)
Supplement: Figures S1 to S3 [file mmc1.pdf]

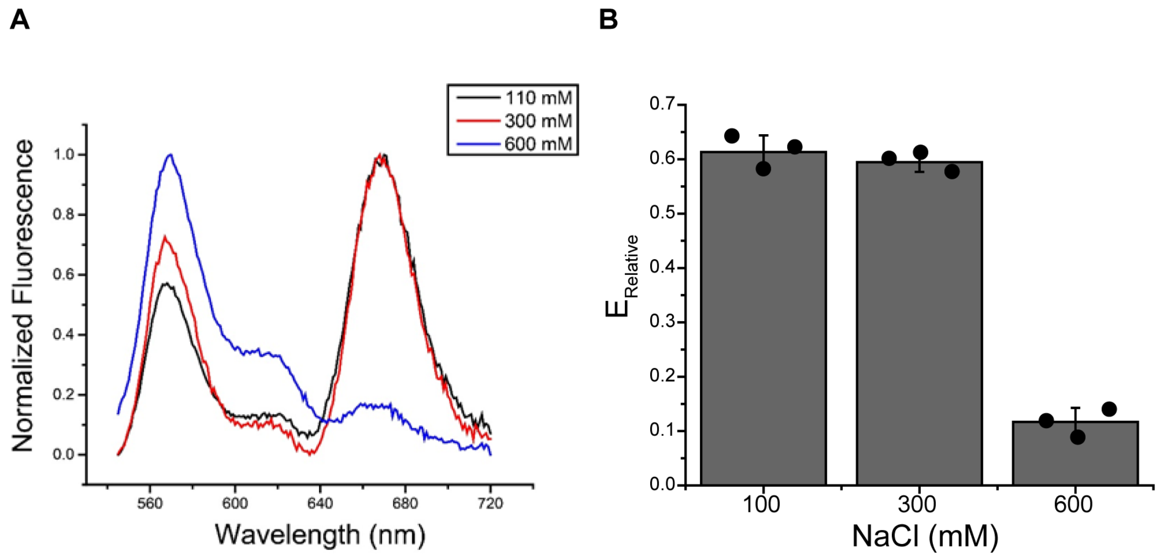

Figure S1. Nucleosome stability in varying NaCl concentrations. 601 nucleosomes were labeled with Cy3 on H2A and Cy5 on the NPS DNA. This labeling strategy was designed to produce high FRET when the NPS DNA is correctly positioned on the histone octamer. (A) Fluorescence spectra of nucleosomes in the presence of 110 mM, 300 mM, or 600 mM NaCl. The Cy3 (570 nm) and Cy5 (670 nm) emission peaks result in identical FRET in the presence of 110 mM and 300 mM NaCl. The FRET signal decreases in the presence of 600 mM NaCl indicating the nucleosomes are no longer intact. (B) Quantitation of the ratiometric FRET ($E_{Relative}$). Black circles indicate values from each experiment. Error bars indicate the standard deviation between three experiments. Nucleosomes were from three independent assemblies.

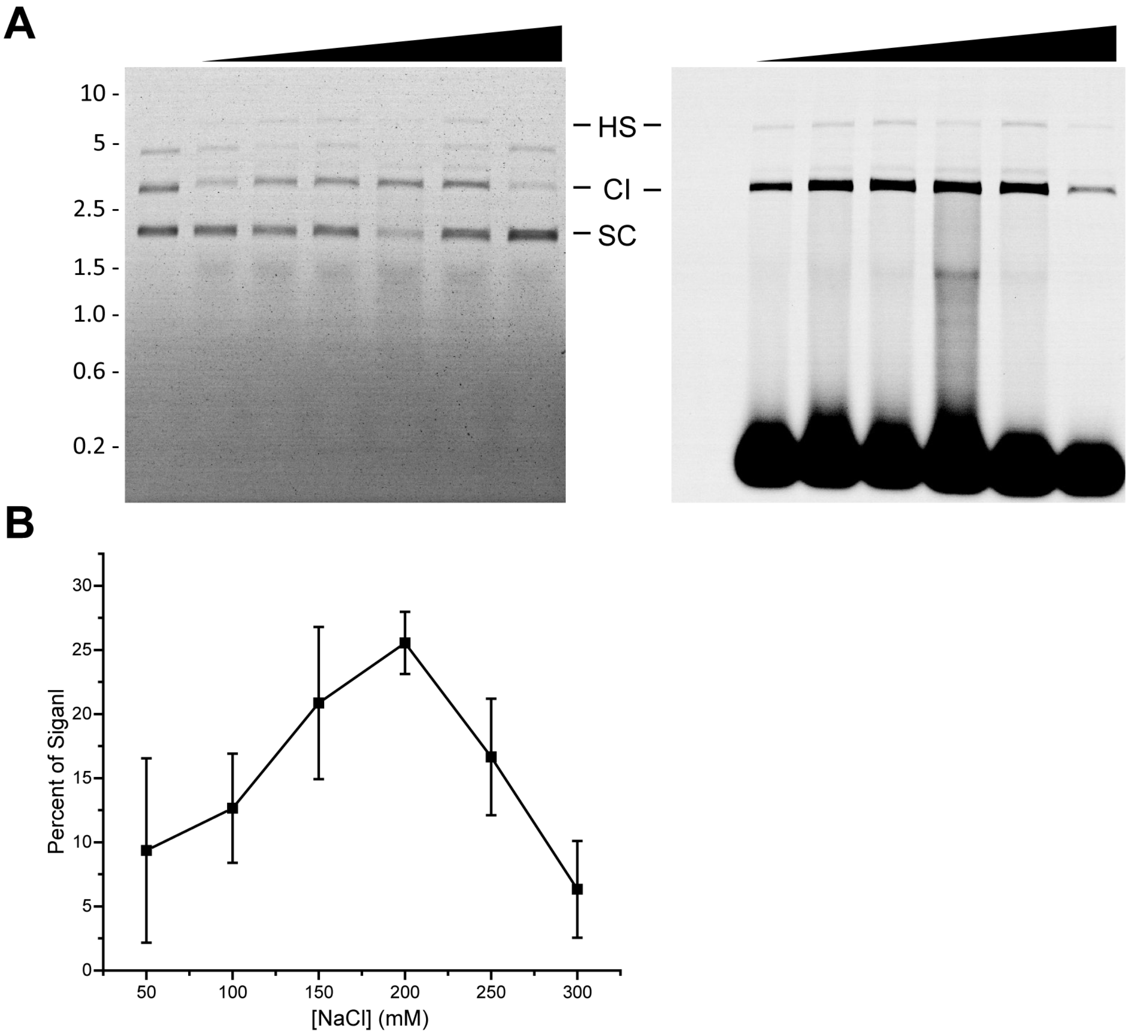

Figure S2. PFV intasomes are active at high salt concentrations. (A) PFV intasomes assembled with Cy5 labeled vDNA were added to 3 kb supercoiled plasmid DNA in the presence of increasing concentrations of NaCl (black triangle, 50, 100, 150, 200, 250, and 300 mM NaCl). The left lane is supercoiled 3 kb plasmid substrate alone. Reaction products were separated by 1% agarose gel electrophoresis. Gels were imaged for ethidium bromide fluorescence (left) or Cy5 fluorescence (right). DNA size marker in kb on the left. Mobilities of half site integration (HS) products, concerted integration (CI) products, and supercoiled plasmid (SC) substrate are indicated. (B) The total PFV integration was calculated from the Cy5 image as the percentage of the total fluorescent signal in the gel. The smear of multiple integration products below the CI band was included. Error bars indicate the standard deviation between at least three independent experiments and at least two independent intasome preparations.

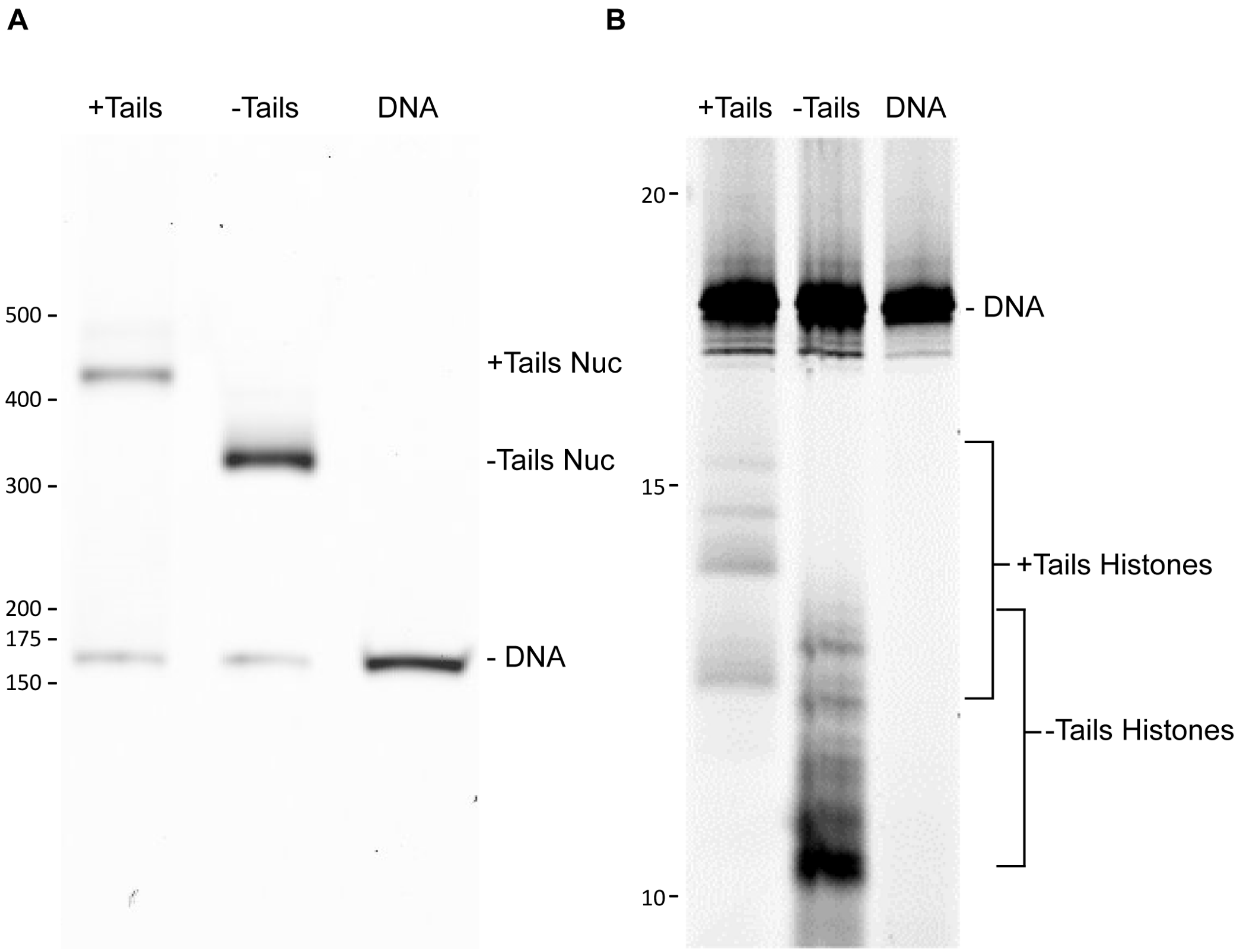

Figure S3. Trypsinization of 601 nucleosomes. 601 nucleosomes with Cy5 labeled NPS DNA were treated with trypsin to remove the histone tails. (A) Untreated nucleosomes (+Tails), trypsin treated nucleosomes (-Tails), and NPS DNA (DNA) were analyzed by native PAGE. The gel was scanned for Cy5 fluorescence. Nucleosomes treated with trypsin (-Tails) display faster mobility than untreated (+Tails). (B) 601 histone proteins were labeled with Cy5 NHS ester. Nucleosomes were treated with trypsin and analyzed by denaturing PAGE. The Cy5 fluorescence image shows the NPS DNA (DNA), histones not digested with trypsin (+Tails), and histones digested by trypsin treatment of the nucleosome (-Tails). Protein molecular weight markers in kD are shown on the left.
